# Prevalence of and factors associated with anemia in school children from Maceió, northeastern Brazil

**DOI:** 10.1186/s12889-016-3073-2

**Published:** 2016-05-10

**Authors:** Haroldo da Silva Ferreira, Myrtis Katille de Assunção Bezerra, Monica Lopes de Assunção, Risia Cristina Egito de Menezes

**Affiliations:** Faculty of Nutrition, Federal University of Alagoas, Campus A.C. Simões, BR 104 Norte - Km 96.7 - Tabuleiro do Martins, CEP: 57072-970 Maceió, AL Brazil; Centro de Pesquisa Aggeu Magalhães, Fundação Oswaldo Cruz, Av. Prof. Moraes Rego, s/n - Campus da UFPE - Cidade Universitária, Recife, PE Brazil

**Keywords:** Anemia, Associated factors, Food consumption, Schools

## Abstract

**Background:**

Anemia is a major public health problem in preschool children in Alagoas, Brazil, especially in the younger population, because of a positive correlation between hemoglobin level and age. However, due to the lack of studies, it is not known how widespread this condition is among older children or how it is associated with socioeconomic status. The aim of this study was to investigate the prevalence of and factors associated with anemia in school children from the city of Maceió, Alagoas state, Brazil. The analysis was stratified to identify any effect modification according to whether they attend public or private schools.

**Methods:**

A cross-sectional study was conducted using probability sampling of 1518 children (9.8 ± 0.5 years of age) attending public (*n* = 931) and private (*n* = 587) elementary schools in the municipality. Semi-structured questionnaires were used to obtain socioeconomic, demographic, anthropomorphic, hemoglobin (Hb), and dietary data. Anemia (Hb <11.5 g/dL) was the dependent variable. The measure of association used was prevalence ratio (PR) and respective CI95 %, calculated by Poisson regression with robust variance adjustment, for the crude and adjusted analyses.

**Results:**

The prevalence of anemia was found to be higher amongst the public school children than the private school children (10.8 % vs. 7.0 %; PR = 1.54; CI95 %: 1.1; 2.2). At the public schools, the variables that remained significantly associated with anemia after the multivariate analysis were “consumption of fruits/fruit juices < 2 portions/day” (PR = 2.19; CI95 %: 1.18; 4.06) and “male” (PR = 1.51; CI95 %: 1.001; 2.30). At the private schools, these variables were “working mother” (PR = 2.83; CI95 %: 1.23; 6.52) and “monthly school fees < R$ 188.00” (PR = 3.20; CI95 %: 1.46; 7.03).

**Conclusions:**

In the population under study, anemia constitutes a major health problem, especially among children from public schools. Considering the associated factors and the fact that these children are in a school environment, the promotion of healthy eating habits could constitute an important approach for reducing the prevalence of anemia.

## Background

The World Health Organization (WHO) defines iron deficiency anemia as a state in which blood hemoglobin concentrations are abnormally low in virtue of the deficiency of one or more essential nutrients, no matter what the cause of this deficiency [1]. Affecting around 25 % of the world population, anemia is currently the most widespread nutritional deficiency worldwide. According to data from a literature review conducted by Mujica-Coopman et al. [2], in Brazil, Guatemala, the Dominican Republic, and Bolivia, anemia in children under 6 years of age is a moderate public health problem whose prevalence ranges from 21.4 to 38.3 %.

Despite its multifactorial etiology and distribution amongst all social strata, anemia is more frequent in contexts characterized by poor socioeconomic conditions, and where there is a low consumption of iron alongside the intake of substances that inhibit iron absorption [3–5]. Thus, although there are several other factors that affect the availability of iron to meet the metabolic requirements of the organism, the risk factors for anemia most frequently cited in the literature are low family income, low maternal level of education, and inadequate iron intake [6, 7]. In Brazil, the short duration of exclusive breastfeeding, diarrhea, and lack of basic sanitation are also reported as major factors associated with low hemoglobin levels [8].

Although pregnant women and children under 5 are the biological groups with the highest susceptibility to iron deficiency anemia, it also affects many school-age children, which is a concern because of the impacts it has on physical and intellectual capacity and on the growth and development of individuals, while also predisposing them to a higher frequency of morbidity [1, 9–11].

As such, the school performance, standard of health, and quality of life of individuals with anemia tend to be lower than in people who are not anemic, for which reason initiatives for its prevention and treatment should be prioritized in the public policies geared towards this age group [12].

This consideration is especially relevant in Alagoas, a Brazilian state with some of the worst social indicators, such as illiteracy rate and per capita family income [13]. Surveys conducted in this state found that 45 % of children aged 6 to 59 months [14] and 50 % of pregnant women [15] were anemic. However, no studies of school-age children have been done in the state.

In Brazil, governments have introduced a number of measures designed to control major endemic nutritional deficiencies: wheat and corn flour fortification with iron; ferrous sulfate supplementation (children aged 6–18 months, pregnant women from the 20th week of gestation, mothers until the 3rd month postpartum); the promotion of breastfeeding; nutritional education; and school feeding programs [16, 17]. However, unlike what has been observed with regard to protein-energy malnutrition, the prevalence of which has dropped significantly in recent years, anemia is just as prevalent or even more so in some specific contexts [18].

Considering its multifactorial etiology, it is important to identify what factors are associated with its occurrence in different epidemiological contexts in order for the best measures to be introduced for each situation [1].

The aim of this study was to investigate the prevalence of and factors associated with anemia in school-age children from the Brazilian city of Maceió, looking for effect modification by stratifying the analysis according to whether they attend public or private schools.

## Methods

A cross-sectional study was carried out between October 2012 and May 2013. The project was approved by the research ethics committee at the Federal University of Alagoas (Process # 017299/2011-43).

The sample consisted of male and female children aged between 9 and 11 years enrolled in elementary schools in Maceió, the state capital of Alagoas, Brazil. This criterion was established to prevent any children under 9 or over 11 being included in the study, in the former case because they would find it harder to answer the questionnaires, and in the latter case because they would be at the early stages of puberty and therefore more susceptible to anemia because of the associated increased dietary iron requirements.

The sample size calculation was conducted using the StatCalc module of the Epi Info™ software tool, version 7.1.3.10. According to the Brazilian Educational Census [19], the study population comprises 31,488 students. In the absence of any other published research on anemia in the target age group, we took the expected prevalence to be the same as that observed for the 48–60 month age group (18.5 %) in a study of preschool children in Alagoas [14]. The other parameters considered in the calculation of sample size were: sampling error of 2.5 percentage points, number of clusters (schools) equal to 80, and a 95 % confidence interval (CI). We also added a design effect of 1.5 to correct the error related to the multistage sample selection process [20], as well as allowing for up to 15 % missing data and refusals. Accordingly, a total sample of 1360 individuals was estimated.

The sample was obtained by two-stage cluster sampling. In the first stage the schools were selected and in the second phase the students in the target age group enrolled in these schools were selected. To ensure geographic homogeneity across the different localities in the municipality, a systematic sample of 80 schools (40 public and 40 private) was obtained from a list of all the educational establishments in the city. To maintain the same proportion of students from each type of school in the sample (2/3 from public and 1/3 from private schools), 26 students should have been selected from each public school and 13 from each private school. However, after the data collection was started, it was found that there were not enough eligible students in all the private schools. To compensate for a possible sample deficit, it was decided that whenever a private school had more students in the target age group, then 16 would be selected rather than 13. With this procedure, the aim was to obtain a sample of 1560 students: 1040 from public schools and 520 from private schools. The students were selected randomly from a list provided by the staff of the respective educational establishments.

A previously trained team collected the data using two strategies. Sociodemographic questions and questions related to the children’s perinatal health were included in a single questionnaire, which was sent via the students to their parents. Completed questionnaires were returned to the researchers, together with informed consent forms. Meanwhile, anthropometric, hemoglobin, dietary, and health data was obtained directly from each of the students using semi-structured questionnaires.

Hemoglobin (Hb) levels were measured using HemoCue®. Anemia (Hb <11.5 g/dL) was classified as mild (11.0 g/dL ≤ Hb ≤11.4 g/dL), moderate (8.0 g/dL ≤ Hb ≤10.9 g/dL), and severe (Hb < 8.0 g/dL) [21].

In the anthropometric analysis, the WHO’s reference data were used [22], while the protocol recommended by Frisancho was followed to obtain the data [23]. Weight was measured using Tanita® HD313 scales (150 kg/100 g). Height was measured using a Seca® stadiometer with a collapsible measuring rod (220 cm/0.1 cm). The data were treated using AnthroPlus [24], which is based on WHO Reference 2007 [22]. Stunting was established as height-for-age < -2 standard deviations (SD). The obesity indicator used was Body Mass Index (BMI)-for-age > 2 SDs. Values below this cut-off point but above 1 SD were classified as overweight (1 < z score ≤ 2). The term “excess weight” was employed to refer to the sum of the cases of overweight and obesity.

For the economic classification, the criteria proposed by ABEP [25] were used, which classifies individuals into classes A1, A2, B1, B2, C1, C2, D, and E according to the total score they are given, with A representing the highest class. The total score is the sum of the scores assigned for the number of durable goods in the child’s household, the level of education of the head of household, the number of toilets in the household, and the number of domestic workers (maids). It was decided not to use the sub-classes, so the children’s households were classified into five economic groups: A, B, C, D, and E. Due to the small number of individuals classified in some of the categories, the three highest categories (A + B + C) were compared with the two lowest categories (D + E) and the analysis was dichotomous.

Still looking at the socioeconomic variables, another factor to be analyzed was whether the school was privately or government-run, and in the case of the private schools, the monthly school fees.

Two categories were used to classify per capita income: ≤ or > US$ 2.00/day/person at the time of the study (US$ 1.00 = R$ 2.14).

Information on skin color/ethnicity was collected by the interviewers through direct observation. For the statistical analysis, the individuals classified as brown, white, oriental, and indigenous were all combined into a single category (“non-black”) and a dichotomous analysis was performed (black vs. non black).

The children’s food consumption was obtained using a Previous Day Food Questionnaire (PDFQ) [26] structured into six meals a day, with each containing graphic representations of 23 food groups. This was used to identify qualitative food intake the day before the interview. The interviews were conducted to obtain the students’ dietary recall from weekdays. When it so happened that an interview was held on a Monday, the students reported on the previous day’s dietary intake (Sunday). However, no interviews were held after holidays to reduce the risk of gathering atypical data [27].

The ingestion of coffee was investigated, as it is believed to have a negative effect on iron absorption, as was the consumption of foods considered to be sources of iron or capable of promoting its absorption (meats, fish, poultry, fruits, fruit juices, vegetables, and beans) [3–5]. The students answered the questionnaires together with a researcher individually in a designated space inside the schools. Intake was considered adequate when the children reported the consumption of (in portions/day): ≥1 of meats; ≥1 of beans; ≥3 of fruits/fruit juices; ≥3 of vegetables; and <1 of coffee. The food data analysis followed the recommendations of the Food Guide for the Brazilian Population [28]. Furthermore, it was assumed that the consumption of one or more portions of coffee a day [3] and <2 portions/day of fruits/fruit juices represented a risk factor for anemia.

The children’s parents/guardians were given a questionnaire designed to investigate the conditions that may influence the children’s health, such as: duration of exclusive breastfeeding (≥6 months/<6 months), prenatal care (yes/no), and birth weight (low <2500 g; high ≥4000 g).

The results were inputted twice into a Google docs® environment. The two databases were compared and the divergences caused by typing errors were corrected. All statistical analysis was conducted with Stata® 12.0 (StataCorp, College Station, Texas).

The sample was characterized using both absolute and relative frequency. To analyze the association between anemia (dependent variable) and the independent variables, the chi-squared test (or Fisher’s exact test for frequencies of five or fewer cases) and prevalence ratio (PR) with respective CI95 % were used as a measure of association.

The associations that yielded *p* < 0.2 in the crude analysis were submitted to multivariate analysis to control any potential confounding factors. However, due to the excessive number of answers missing on the questionnaires returned by the parents, some variables were not included in the multivariate analysis, namely: birth weight, household income, number of household members, access to prenatal care, and duration of exclusive breastfeeding.

The PRs and their CI95 % were calculated by Poisson regression analysis with robust variance adjustment, in both crude and adjusted analysis. The level of statistical significance for variables to remain in the final model was set at 0.05. Marginal statistical significance was the term used when 0.05 ≤ *p* <0.1.

## Results

At the private schools, 638 children were identified who were eligible for the study. Of these, 587 (92.2 %) were studied; the remaining 51 (7.9 %) were not investigated because they did not return the informed consent form signed by a parent or guardian. 931 children from public schools were studied from the total of 960 eligible students (29 refusals; 3.0 %).

The general prevalence of anemia was found to be 9.3 %, but the value for the public school children was higher than it was for the private school children (10.8 % vs. 7.0 %; PR = 1.54; CI95 %: 1.1; 2.2). 4.5 % of the public school children were found to have moderate anemia, while this figure was 2.2 % for the private school children. Two children, both from public schools, were diagnosed with severe anemia.

Table 1 shows some of the characteristics of the sample. It can be seen that there was a higher proportion (*p* < 0.05) of children from public schools than from private schools from classes D and E, meaning they have lower economic means (55.6 % vs. 11.4 %); with women as heads of household (43.9 % vs. 30.2 %); of black skin color (17.0 % vs. 4.6 %); with unemployed parents (29.5 % vs. 6.4 %); and from a household with six individuals or more (24.9 % vs. 10.9 %).

**Table 1:** Socioeconomic, demographic and anthropometric characteristics of the elementary school children, broken down by whether they study at public or private schools. Maceió, Alagoas, Brazil, 2013

Variables	Total n (%)	Type of school		*p*
		Private n (%)	Public n (%)	
Economic class				
A	15 (1.4)	15 (2.9)	0 (0.0)	
B	205 (19.3)	192 (36.4)	13 (2.4)	
C	485 (45.6)	260 (49.3)	225 (42.0)	
D	333 (31.3)	57 (10.8)	276 (51.5)	
E	25 (2.4)	3 (0.6)	22 (4.1)	
D + E	358 (33.7)	60 (11.4)	298 (55.6)	<0,001^a^
Head of household (sex)				
Male	531 (62.8)	287 (69.8)	244 (56.1)	
Female	315 (37.2)	124 (30.2)	191 (43.9)	<0.001
Father in work				
Yes	708 (82.3)	411 (93.6)	297 (70.5)	
No	152 (17.7)	28 (6.4)	124 (29.5)	<0,001
Number of household members				
<6	852 (82.2)	466 (89.1)	386 (75.1)	
≥ 6	185 (17.8)	57 (10.9)	128 (24.9)	<0,001
Color of skin / Ethnicity				
Brown	812 (56.2)	264 (46.5)	548 (62.4)	
White	427 (29.5)	261 (46.0)	166 (18.9)	
Black	175 (12.1)	26 (4.6)	149 (17.0)	<0.001^b^
Oriental	28 (1.9)	15 (2.6)	13 (1.5)	
Indigenous	4 (0.3)	2 (0.3)	2 (0.2)	
Classification of nutritional status by BMI-for-age				
Overweight (1 < z ≤ 2)	253 (18.3)	117 (20.8)	136 (16.6)	
Obese (z > 2)	213 (15.4)	125 (22.2)	88 (10.7)	<0,001
Excess weight (z > 1)	466 (33.7)	242 (43.0)	224 (27.3)	
Classification of nutritional status by height-for-age				
Stunting (z < -2)	24 (1.7)	4 (0.7)	20 (2.3)	0.016
Birth weight (BW)				
Low (BW < 2500 g)	75 (8.4)	31 (6.7)	44 (10.3)	0.051
High (BW ≥ 4000 g)	69 (7.7)	43 (9.2)	26 (6.1)	0.078

N.B. The sum of the total number varies because of data gaps

^a^A dichotomous analysis was conducted of the highest categories (A + B + C) compared with the lowest categories (D + E)

^b^A dichotomous analysis was conducted comparing “black” with the sum of the other categories (brown, white, oriental, and indigenous)

Tables 2 and 3 show the distribution of anemia according to the independent variables and the type of school. It was found that “working mother” was a risk factor for anemia in children from private schools (PR = 2.52; CI95 %: 1.11; 5.68).

**Table 2:** Prevalence of anemia, prevalence ratio (PR) and respective confidence interval at 95 % for selected variables amongst children from public elementary school in Maceió, Alagoas, Brazil, 2013

Variables	Total n (%)	Anemia n (%)	PR (CI95 %)	*p*
Sex				
Female	461 (51.9)	39 (8.5)	1	0.019*
Male	427 (48.1)	57 (13.3)	1.57 (1.07; 2.32)	
Duration of exclusive breastfeeding				
≥ 6 months	156 (51.0)	16 (10.3)	1	0.786
< 6 months	150 (49.0)	14 (9.3)	0.91 (0.45; 1.80)	
Prenatal care				
Yes	481 (84.4)	47 (9.7)	1	0.095
No	89 (15.6)	14 (15.7)	1.61 (0.92; 2.79)	
Employment status of father				
Employed (formal / informal)	294 (71.2)	23 (7.8)	1	0.077
Unemployed	119 (28.8)	16 (13.5)	1.71 (0.94; 3.13)	
Employment status of mother				
Unemployed	328 (60.4)	34 (10.4)	1	0.769
Employed (formal / informal)	215 (39.6)	24 (11.2)	1.07 (0.63; 1.83)	
Number of household members				
< 6	375 (74.8)	33 (8.8)	1	0.026*
≥ 6	126 (25.1)	20 (15.9)	1.80 (1.07; 3.02)	
Per capita income				
>2.00 dollars/day	243 (60.3)	20 (8.2)	1	0.161
≤2.00 dollars/day	160 (39.7)	20 (12.5)	1.51 (0.84; 2.73)	
Recipient of government benefits				
No	127 (23.1)	14 (11.0)	1	0.795
Yes	411 (76.4)	42 (10.2)	0.92 (0.52; 1.64)	
Height-for-age				
≥ −2 z (eutrophic)	800 (97.5)	83 (10.4)	1	0.957
< −2 z (stunted)	20 (2.5)	2 (10. 0)	0.96 (0.25; 3.64)	
BMI-for-age				
≤ 2 z (eutrophic)	704 (89.4)	76 (10.8)	1	0.088
> 2 z (obese)	83 (10.5)	4 (4.8)	0.44 (0.16; 1.18)	
Consumption of meat, fish or poultry				
≥1 portions	773 (87.2)	84 (10.9)	1	0.937
0 portions	113 (12.8)	12 (10.6)	0.97 (0.55; 1.71)	
Consumption of fruits/fruit juices				
≥3 portions	80 (9.0)	4 (5.0)	1	0.078
<3 portions	805 (91.0)	92 (11.4)	2.28 (0.86; 6.05)	
Consumption of fruits/fruit juices				
≥2 portions	242 (27.3)	15 (6.2)	1	0.006*
<2 portions	643 (72.6)	81 (12.6)	2.03 (1.19; 3.45)	
Consumption of coffee				
0 portions	412 (46.4)	36 (8.7)	1	0.064
≥ 1 portions	476 (53,6)	60 (12,6)	1,89 (1,00; 1,4)	

*indicates statistical significance (*p* < 0.05); *PR* prevalence ratio, *CI95 %* confidence interval 95 %

N.B. the sum of the total number varies because of data gaps

**Table 3:** Prevalence of anemia, prevalence ratio (PR) and respective confidence interval at 95 % for selected variables amongst children from private elementary school in Maceió, Alagoas, Brazil, 2013

Variables	Total n (%)	Anemia n (%)	PR (CI95 %)	*p*
Sex				
Female	283 (50.8)	20 (7.1)	1	0.951
Male	274 (49.2)	19 (6.9)	0.98 (0.53;1.79)	
Duration of exclusive breastfeeding				
≥ 6 months	185 (46.7)	7 (3.8)	1	
< 6 months	211 (53.3)	22 (10.4)	2.75 (1.20;6.30)	0.011*
Prenatal care				
Yes	492 (96.7)	32 (6.5)	1	
No	17 (3.3)	2 (11.8)	1.86 (0.44;7.82)	0.393
Employment status of father				
Employed (formal / informal)	399 (93.4)	25 (6.3)	1	
Unemployed	28 (6.6)	2 (7.1)	1.14 (0.28;4.57)	0.854
Employment status of mother				
Unemployed	202 (39.5)	27 (8.7)	1	
Employed (formal / informal)	309 (60.5)	7 (3.5)	2.52 (1.11;5.68)	0.019*
Number of household members				
< 6	450 (88.9)	29 (6.4)	1	
≥ 6	56 (11.1)	4 (7.14)	1.1 (0.40;3.03)	0.842
Per capita income				
>2.00 dollars/day	386 (96.3)	25 (6.5)	1	
≤2.00 dollars/day	15 (3.7)	1 (6.7)	1.02 (0.14; 7.11)	0.977
Recipient of government benefits				
No	406 (78.1)	26 (6.4)	1	
Yes	114 (21.9)	8 (7.0)	1.09 (0.50; 2.35)	0.815
School fees (R$)				
≥188.10	263 (49.1)	11 (4.2)	1	
<188.00	273 (50.9)	26 (9.5)	2.27 (1.14; 4.51)	0.015*
Height-for-age				
≥ −2 z (eutrophic)	550 (99.3)	38 (6.9)	1	
< −2 z (stunted)	4 (0.7)	1 (25.0)	3.61 (0.64; 20.33)	0.159
BMI-for-age				
≤ 2 z (eutrophic)	416 (77.8)	28 (6.7)	1	
> 2 z (obese)	119 (22.2)	10 (8.4)	1.24 (0.62; 2.49)	0.531
Consumption of meat, fish or poultry				
≥ 1 portion	477 (86.7)	35 (7.3)	1	
0 portions	73 (13.3)	4 (5.5)	0.74 (0.27; 2.04)	0.565
Consumption of fruits/fruit juices				
≥ 3 portions	117 (21.3)	4 (3.4)	1	
< 3 portions	433 (78.7)	35 (8.1)	2.36 (0.85; 6.52)	0.081
Consumption of fruits/fruit juices				
≥ 2 portions	219 (42.9)	12 (5.2)	1	
< 2 portions	292 (57.1)	27 (8.5)	1.62 (0.84; 3.14)	0.140
Consumption of coffee				
0 portions	367 (65.9)	26 (7.1)	1	
≥ 1 portion	190 (34.1)	13 (6.8)	0.97 (0.61; 1.54)	0.915

*Indicates statistical significance (*p* < 0,05); *PR* Prevalence ratio, *CI95 %* confidence interval 95 %

N.B. the sum of the total number varies because of data gaps

No statistically significant difference was observed between the prevalence of anemia in black and non-black children from public (*p* = 0.548) or private (*p* = 0.324) schools. In the private schools, a similar level of anemia was found for the girls and the boys, but at the public schools the prevalence of anemia was higher amongst the boys (13.3 % vs. 8.5 %; PP = 1.5; CI95 %: 1.07; 2.32).

At the private schools, 20.8 % of the children were overweight, and 22.2 % were obese (43.0 % with excess weight), while 0.7 % were stunted. At the public schools, the prevalence of overweight and obesity was 16.5 and 10.7 %, respectively (27.2 % with excess weight), while 2.3 % of the children were found to be stunted. No significant association was found between anemia and these nutritional conditions.

The average birth weight of the private school children was 3297 g ± 619 g, with a 6.7 % prevalence of low birth weight and 9.9 % prevalence of high birth weight (≥4000 g). Meanwhile, the average weight at birth of the public school children was 3227.0 g ± 603 g, with a prevalence of low and high birth weight of 10.3 and 6.7 %, respectively (Table 1). A marginally statistically significant association was found for the mothers of the public school children who did not have prenatal care, suggesting that this variable represents a certain risk for the development of anemia (*p* = 0.09) (Table 2).

Only amongst the private school children was exclusive breastfeeding < 6 months a risk factor for anemia (10.4 % vs. 3.8 %; PR = 2.75; CI95 %: 1.2; 6.3).

Table 4 shows the information about the consumption of different foods by the public and private school children. The public school children reported consuming more coffee than the private school children (p < 0.001): 21.2 % of them said they drank coffee with two meals a day, and 30.7 % said they took coffee with one meal a day. Amongst the private school children, only 10.1 % said they consumed coffee with two meals a day, and 23.3 % consumed it with one meal. A marginal association was found between higher coffee consumption and a higher prevalence of anemia for the public school children but not for the private school children (12.6 % vs. 6.8 %; *p* = 0.06).

**Table 4:** Characteristics of food consumed by elementary school children, broken down by whether they study at public or private schools. Maceió, Alagoas, Brazil, 2013

Recommendation Portions/Day	n (%)	Type of school		PR (CI95 %)	*p*	Anemia	
		Public n (%)	Private n (%)			Public school n (%)	Private school n (%)
Coffee							
0 portions/day	820 (54.1)	432 (46.5)	388 (66.1)	–	<0.001*	36 (8.7)	26 (7.1)
≥1 portion/day	696 (45.9)	497 (53.3)	199 (33.9)	1.57 (1.38; 1.79)		60 (12.6)	13 (6.8)
						*p* = 0.06	*p* = 0.91
Meat, fish, poultry							
≥1 portion/day	1303 (86.9)	808 (87.2)	501 (86.4)	–	0.623	84 (10.9)	35 (7.3)
0 portions/day	197 (13.1)	118 (12.7)	79 (13.6)	0.93 (0.71; 1.20)		12 (10.6)	4 (5.5)
						*p* = 0.94	*p* = 0.56
Fruit, Fruit Juices							
≥ 3 portions/day	205 (13.6)	83 (9.0)	122 (21.1)	–	<0.001*	4 (5.0)	4 (3.4)
< 3 portions/day	1300 (86.4)	842 (91.0)	458 (78.9)	1.15 (1.10; 1.20)		92 (11.4)	35 (8.1)
						*p* = 0.07	*p* = 0.08
Vegetables							
≥ 3 portions/day	42 (2.8)	17 (1.8)	25 (4.3)	–	0.01*	2 (9.5)	1 (5.26)
< 3 portions/day	1463 (97.2)	908 (98.2)	555 (95.69)	1.02 (1.00; 1.04)		94 (10.8)	38 (7.16)
						*p* = 0.84	*p* = 0.75
Beans							
≥ 1 portion/day	963 (69.5)	616 (72.1)	347 (65.5)	–	0.009**	77 (12.0)	27 (7.5)
0 portions/day	422 (30.5)	239 (27.9)	183 (34.5)	0.81 (0.69; 0.94)		19 (7.6)	12 (6.0)
						*p* = 0.05	*p* = 0.51

*indicates statistical significance (*p* < 0.05); **indicates marginal statistical significance (*p* < 0.1)

N.B. the sum of the total number varies because of data gaps

No significant difference (*p* = 0.62) was found between the public and private school children’s consumption of beans, fish, and poultry. There was no association between the adequate or inadequate intake of these food groups and anemia.

More private school children consumed ≥ 3 portions of fruits/fruit juices (21.1 %); only 9.0 % of the children from the public schools said they consumed this quantity. Few vegetables were consumed by either group, but again the consumption by the public school children was lower (1.8 % vs. 4.3 %; *p* = 0.01).

Beans were on the menu of 72.1 and 65.5 % of the children from public and private schools, respectively.

To test the independence of the associations with anemia in private school children, the following variables were subject to multivariate analysis: “working status of mother,” “height-for-age,” “monthly school fees,” and “consumption of fruits/fruit juices.” After this treatment, “working mother” (PR = 2.83; CI95 %: 1.23; 6.52) and “monthly school fees < R$188.00” (PR = 3.2; CI95 %: 1.46; 7.03) were still significantly associated with anemia. For the public school children, the variables inputted into the model were “sex,” “BMI-for-age,” “consumption of fruits/fruit juices,” and “coffee consumption.” The variables found to be independently associated with anemia were “consumption of fruits/fruit juices <2 portions/day” (PR = 2.19; CI95 %: 1.18; 4.06) and “male” (PR = 1.51; CI95 %: 1.001; 2.30). The other variables introduced to the adjusted model lost their statistical significance (Table 5).

**Table 5:** Adjusted prevalence ratio (PR) and respective confidence interval at 95 % according to selected variables for private and public school children in elementary education in Maceió, Alagoas, Brazil, 2013

Type of school	Variables	PR (CI95 %)*	*p*
Private			
	Employment status of mother		
	Employed (formal/informal)	1	
	Unemployed	2.83 (1.23;6.52)	0.014**
	Monthly school fees (R$)		
	≥188.1	1	
	<188.0	3.20 (1.46; 7.03)	0.004**
	Height-for age (z score)		
	≥ −2 (eutrophic)	1	
	< −2 (stunted)	2.75 (0.73; 10.38)	0.134
	Consumption of fruits/fruit juices		
	≥ 2 portions	1	
	< 2 portions	1.62 (0.78; 3.34)	0.188
Public			
	Sex		
	Female	1	
	Male	1.51 (1.001; 2.30)	0.049**
	BMI-for-age (z score)		
	≤ 2 (eutrophic)	1	
	> 2 (obese)	0.47 (0.17; 1.24)	0.129
	Consumption of fruits/fruit juices		
	≥ 2 portions	1	
	< 2 portions	2.19 (1.18; 4.06)	0.013**
	Consumption of coffee		
	0 portions	1	
	≥ 1 portion	1.27 (0.83; 1.95)	0.267

*PR adjusted for all the variables included in the model; **Indicates statistical significance (*p* < 0.05)

## Discussion

The results presented here indicate that anemia is a public health problem for school-age children in the municipality of Maceió, Brazil. The prevalence rates reported in this study are unacceptable because of the damage this nutritional deficiency can cause to growth and cognitive development in children [29]. A study conducted ten years ago in Maceió [30] found the prevalence of anemia to be very similar to today’s level (9.9 % vs. 9.3 %), showing that the situation has not changed in this period of time.

There is no research in Brazil about the time trends of the prevalence of anemia involving representative samples of school-age children. However, a study of 1108 preschool children from the state of Paraíba found that anemia in that state is still a major public health problem and that little progress has been made in reducing its levels [18]. The authors commented that their expectations of a downward trend in this nutritional deficiency, reflecting the trend in other nutritional deficiencies, especially protein-energy malnutrition, were confounded by their findings of its unaltered status. In this respect, discussions [31, 32] have been conducted about the speed of the rise in the prevalence of obesity while high frequencies of specific nutritional deficiencies are maintained. A potential explanation for this is the adoption across the population of energy-dense but nutrient-poor foods [4, 33].

It could be that the individuals diagnosed with anemia in our research had been anemic since their preschool years, given that their exposure to the different risk factors, like lack of access to an adequate diet and/or the acquisition of constant parasitic infections, would have remained unaltered [17, 34]. However, the cross-sectional design adopted here only picks up the associated factors; it cannot establish causal relationships because it cannot identify the temporal sequence between exposure and the subsequent development of the disease. Indeed, we should add that our findings indicate a positive association between inadequate diet and anemia. Longitudinal studies could shed more light on the continued prevalence of anemia from early life to school age, exacerbating the damage to health caused by this nutritional deficiency.

Looking at the children from public schools, anemia was found to be more prevalent amongst the boys. It would seem that the greater risk of anemia amongst boys is due to their increased weight gain, differences in their food intake, lower iron reserves, higher intestinal iron loss, lower iron absorption, and a higher number of episodes of infection [35, 36]. It is possible that the higher prevalence observed in the pre-adolescent boys is because of factors that influence the occurrence of anemia in childhood and which remain in this age group, which is usually before the onset of puberty, when the situation is reversed. In adolescence, anemia is more prevalent amongst girls because their dietary intake of iron is insufficient to meet the higher iron requirement caused by their growth phase prior to menarche [37, 38].

Amongst the private school children, no association was found between sex and anemia. Perhaps in these households, with higher means, and at this age group [21], the susceptibility to anemia by sex is inverted because in the subgroup in question the onset of puberty is earlier than it is amongst the girls from the public schools because of differences in their body composition. It is an accepted fact that menarche occurs at an earlier age in girls with higher body fat levels than in those with a lower proportion of body fat [39]. In the sample under study here, the private school children had an average z score of BMI-to-age that was higher than that of the children from the public schools (0.56 ± 0.38 vs. 0.15 ± 1.28; *p* < 0.001). There was also a significant difference in the proportion of obese students in the private schools (BMI-age >2 SD): 16.4 % vs. 9.1 %; *p* = 0.003.

Across the board, the adequate consumption of fruits/fruit juices was low, but it was lower for the public school children (9.0 %) than it was for the students at private schools (21 %). Both these figures are lower than the figures found in the southern Brazilian state of Florianópolis [40], where an adequate intake was reported by 27.2 % of the sample. Such low consumption of fruits/fruit juices is unfavorable for the children’s health, increasing the risk of anemia and other conditions [41]. As has already been demonstrated, the prevalence of anemia amongst the children from public schools who consumed inadequate quantities of fruits and juices was 2.2 times as high as the prevalence in the 10 % of the public school children who reported an adequate intake.

The Food Guide for the Brazilian Population recommends the consumption of three portions of fruit daily [28]. However, no significant association was found in this study with this criterion, perhaps because of the low number of subjects who complied with this recommendation (21.1 % of public school children and 9.0 % of private school children). In view of this, we chose to use two or more portions as the criterion in this study, since even this level of consumption was capable of demonstrating a protective effect against anemia.

The determinate effect of household income in anemia seems to derive from the broad effect it has on the qualitative and quantitative availability of food [12]. In this investigation, alongside the higher prevalence of anemia observed amongst the public school children, it was also found that the prevalence amongst the children who went to less expensive private schools was almost twice as high as the prevalence amongst those who attended more expensive private schools.

In view of this association, it would be plausible to assume that having a working mother would help reduce the chance of anemia [42]. However, our study found a higher prevalence of anemia amongst the private school children whose mothers had jobs. It is possible that in these circumstances the mothers have less time to devote to their children. One study on nutrition and socioeconomic conditions found that working mothers were less likely to offer their children an adequate diet [43].

One limitation of this study was the high proportion of parents/guardians who failed to answer the questionnaires sent to them, especially whose children were at public schools (34.2 %). This proportion was lower for the parents of children at private schools (7.1 %). One explanation for this could be the high levels of illiteracy amongst adults in the lowest socioeconomic strata of Maceió. As a consequence of this data gap, some variables found to be associated significantly with anemia in the crude analysis (number of household members ≥6; exclusive breastfeeding <6 months) could not be submitted to the multivariate analysis because it would compromise the adjustment of the model.

In spite of the high proportion of students from public schools whose parents did not return the questionnaire (sociodemographic questions and issues related to the perinatal), we believe that these gaps did not induce a selection bias. The prevalence of anemia amongst the children whose parents failed to return the questionnaire was 10.7 %, while it was 8.2 % among the children whose parents did (*p* = 0.10). These data are in line with the findings of this paper, demonstrating an association between greater social vulnerability and the occurrence of anemia.

The diagnosis of anemia in this study was based exclusively on the measurement of hemoglobin, a fact which constitutes another limitation of this research. Having other hematologic measures in addition to hemoglobin would strengthen the argument that the children’s anemia was related to iron deficiency and help identify the best factors to target in order to reduce the risk of anemia. At the population level, according to Balarajan et al. [44], hemoglobin concentration is the most common indicator, because it is inexpensive and easy to measure with field-friendly testing. However, it lacks specificity for establishing iron status. Therefore, serum ferritin and transferrin receptor concentrations have been recommended as measures of the iron status of populations.

In this study, there were almost 2.5 times as many public school children living in households with six or more individuals than there were private school children. The prevalence of anemia amongst the children in such households was 80 % higher than it was for the children living in households with fewer than six individuals. Higher numbers of people living under the same roof indicates socioeconomic vulnerability and therefore increased food insecurity [45].

Inadequate diet was an important factor related to anemia in this study. However, the use of a non-quantitative food intake evaluation method (PDFQ) could have compromised the association analysis. According to Burrows et al. [46], it is a widely held consensus that there is no perfect method of assessing dietary intake. In the case of children, the inherent difficulties are compounded by their level of cognitive development, capacity to concentrate, and ability to recall foods and estimate portion sizes. In this study, to reduce the possible bias caused by such factors, we limited the sample to children aged around 10. Despite the possible limitations, our findings tend to corroborate those of other studies [40, 47].

The diet of the public school children was found to be quite different than that of their private school peers, with coffee consumption by the public school children constituting one such difference. It has been found that children from poorer households tend to consume more coffee than children from higher social classes. According to Antunes et al. [48], the higher the food insecurity, the greater the coffee consumption. It is a drink that contains high concentrations of polyphenols, which may reduce iron absorption by up to 85 % [3].

Currently, it is understood that not only anemia but also slight or moderate iron deficiency may have an adverse effect on cognitive development, marked by a loss of concentration, sleepiness, and irritability, which can hinder academic performance. Furthermore, immunological capacity is compromised, facilitating the occurrence and/or severity of infectious diseases [1, 12, 49].

## Conclusions

In the population under study, anemia constitutes a major health problem, especially among children from public schools. The significant risk factors were found to be low consumption of fruits, and studying at a public school or a private school with low school fees.

Considering the associated factors and the fact that these children are in a school environment, the promotion of healthy eating habits could constitute an important approach for reducing the prevalence of anemia.

It is recommended that public policy managers pay closer attention to these findings, in view of the associated damage to health, cognitive development, and the quality of life of the individuals affected.

## Ethics approval and consent to participate

The research protocol was approved by the Ethics Committee of the Federal University of Alagoas (Process # 017299/2011-43). Only children who returned informed consent forms in writing duly signed by their respective guardians were included in the study.

## Consent to publish

Not applicable.

## Availability of data and materials

The database can be shared by the corresponding author upon request.

## Abbreviations

95 % CI: 95 % confidence intervals
ABEP: Associação Brasileira de Empresas de Pesquisa
BMI: Body Mass Index
Hb: hemoglobin
PDFQ: Previous Day Food Questionnaire
PR: prevalence ratio
SD: standard deviations
WHO: World Health Organization
